# Initial Experience of the Synchronized, Real-Time, Interactive, Remote Transthoracic Echocardiogram Consultation System in Rural China: Longitudinal Observational Study

**DOI:** 10.2196/14248

**Published:** 2019-07-08

**Authors:** Luwen Liu, Shaobo Duan, Ye Zhang, Yuejin Wu, Lianzhong Zhang

**Affiliations:** 1 People's Hospital of Zhengzhou University Zhengzhou China; 2 Henan Provincial People’s Hospital Zhengzhou China

**Keywords:** three synchronization, double-real-time, interactive, remote consultation on UCG, dynamic image decoding, synchronization technology

## Abstract

**Background:**

China has a vast territory, and the quality of health care services provided, especially transthoracic echocardiography (TTE), in remote regions is still low. Patients usually need to travel long distances to tertiary care centers for confirmation of a diagnosis. Considering the rapid development of high-speed communication technology, telemedicine will be a significant technology for improving the diagnosis and treatment of patients at secondary care hospitals.

**Objective:**

This study aimed to discuss the feasibility and perceived clinical value of a synchronized, real-time, interactive, remote TTE consultation system based on cloud computing technology.

**Methods:**

By using the cloud computing platform coupled with unique dynamic image coding and decoding and synchronization technology, multidimensional communication information in the form of voice, texts, and pictures was integrated. A remote TTE consultation system connecting Henan Provincial People’s Hospital and two county-level secondary care hospitals located 300 km away was developed, which was used for consultation with 45 patients.

**Results:**

This remote TTE consultation system achieved remote consultation for 45 patients. The total time for consultation was 341.31 min, and the mean time for each patient was 7.58 (SD 6.17) min. Among the 45 patients, 3 were diagnosed with congenital heart diseases (7%) and 42 were diagnosed with acquired heart diseases (93%) at the secondary care hospitals. After expert consultation, the final diagnosis was congenital heart diseases in 5 patients (11%), acquired heart disease in 34 patients (76%), and absence of heart abnormalities in 6 patients (13%). Compared with the initial diagnosis at secondary care hospitals, remote consultation using this system revealed new abnormalities in 7 patients (16%), confirmation was obtained in 6 patients (13%), and abnormalities were excluded in 6 patients (13%). The expert opinions agreed with the initial diagnosis in the remaining 26 patients (58%). In addition, several questions about rare illnesses raised by the rural doctors at the secondary care hospitals were answered.

**Conclusions:**

The synchronized real-time interactive remote TTE consultation system based on cloud computing service and unique dynamic image coding and decoding technology had high feasibility and applicability.

## Introduction

Transthoracic echocardiography (TTE) can help doctors visualize the structure, geometric morphology, spatial relationship, motion, and blood flow status of the cardiac chambers intuitively, accurately, and comprehensively [1]. With recent improvements in the spatial and temporal resolution of TTE, TTE has become a routine method for the diagnosis, treatment, and prognostic prediction of cardiovascular diseases [2]. However, image collection and diagnostic processes of TTE are different from those of other imaging technologies that rely on echocardiographers. Echocardiographers are required not only to master the basic theories and knowledge about TTE and heart diseases, but also be skilled in collecting standard images from multiple angles and on multiple planes using TTE [3]. Therefore, TTE-based diagnosis is still challenging for echocardiographers at secondary care hospitals.

Telemedicine is a multidisciplinary approach that integrates modern communication, electronic technology, computer network, and medical science. It can be further divided into remote consultation of patients, remote imaging, remote electrocardiogram, remote pathology, and remote ultrasound consultation and diagnosis. The development of telemedicine covered four major stages, namely, germination, simulation, digital transmission, and integration [4]. Remote TTE consultation was first reported by Canadian doctors Finley et al [5] in the 1980s, who sent audio and video signals in the form of microwaves to pediatric heart disease experts 500 miles away for interpretation and diagnosis using audio and video transmission technology. Along with the development of communication technology, the United States and Western Europe later reported the use of an integrated service digital network and a T1 line for remote TTE service in real-time, storage, and upload modes [6]. In recent years, many reports on internet-based remote consultation have been published. In 2013, physicians Webb et al [7] from Washington DC performed a multicenter study, which demonstrated the potentials of using remote consultation to spare patients from long-distance travelling, saving time and costs while increasing the quality of medical service.

China has a vast territory, and the health care service in remote regions is still poor. Although there have been programs for couplet assistance, medical treatment combination, and disciplinary alliance between tertiary care centers and secondary care hospitals, telemedicine remains the major solution for construction of a hierarchical medical system. At present, telemedicine services already provided are generally remote clinical consultation, remote imaging, remote TTE, and remote pathology consultation and diagnosis. Remote ultrasound consultation, especially remote TTE, is relatively rare due to technical limitations. A synchronized real-time interactive remote TTE consultation system based on cloud computing technology has been jointly built by Henan Provincial People’s Hospital and two county-level secondary care hospitals 300 miles away. The preliminary results achieved with this system are provided in the Results.

## Methods

### Recruitment

A remote TTE consultation platform was built between our hospital and two secondary care hospitals 300 km away, namely, Fanxian People’s Hospital of Puyang City and Zhenping County People’s Hospital of Nanyang City. All participants of the present study signed the informed consent. The confirmed diagnosis of all these patients was not made by TTE at the secondary care hospitals. In order to save time and costs of the patients, TTE consultation was applied by the secondary care hospitals. From September to December 2018, 45 patients were recruited and received remote TTE consultation.

### Equipment

The two secondary care hospitals were equipped with TTE probes and devices with built-in software (ACUSON Oxana 2, Siemens, Berlin/Munich, Germany; Resona 8, Mindray, Shenzhen, China), dynamic image decoders, desktop computers, camera lens, microphones, and broadband network (≥4M). The remote consultation center of Henan Provincial People’s Hospital was equipped with a computer terminal, camera lens, and broadband network.

### Connection and Functional Realization

**Figure 1:**
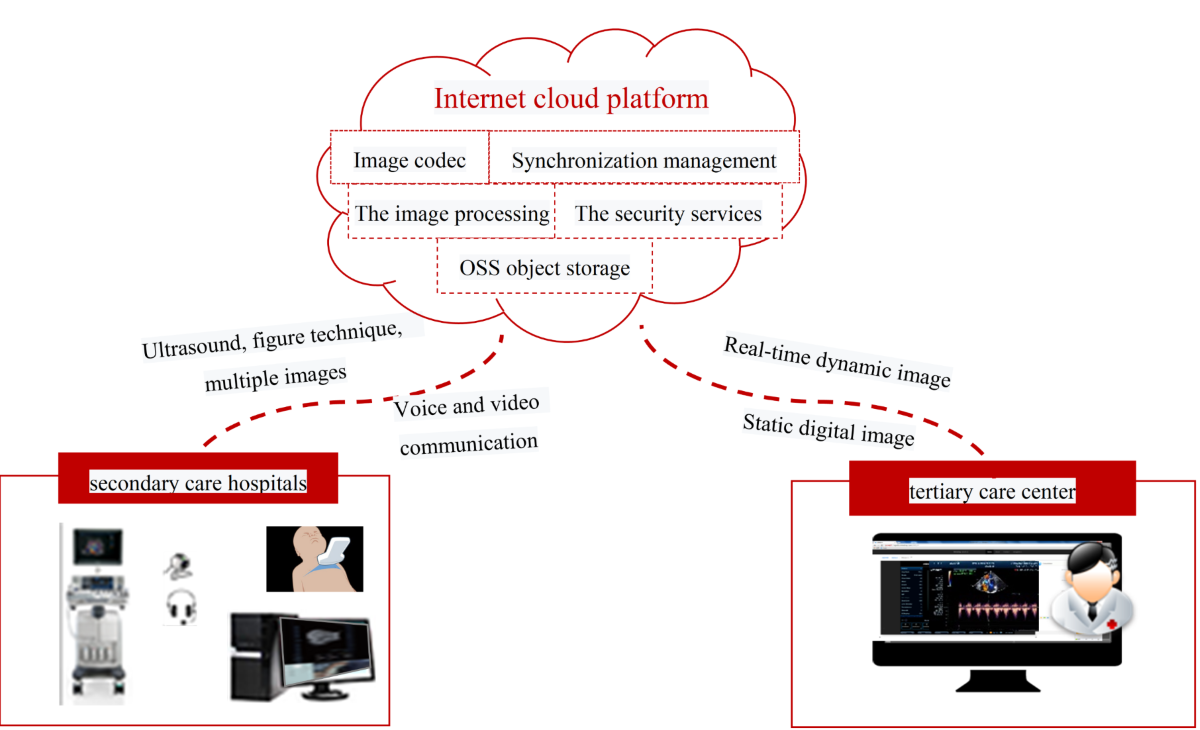
Architecture and workflow of the remote transthoracic echocardiography system. The left and right inserts show the equipment required for remote consultation at the secondary care hospitals and the tertiary care centers, respectively. The transthoracic echocardiography images along with the audio and video information are transmitted via the internet-based cloud platform. OSS: object storage service.

The remote consultation workstation was connected to the TTE device via a dynamic image decoder and HDMI (high-definition multimedia interface) cable for image collection and transmission. The camera lens captured the real-time operation screen of echocardiographers at the secondary care hospitals. The microphone was connected for audio collection. The signals from various channels were sent to the cloud after processing by the workstation. At the other end of consultation, dynamic TTE images were acquired from the cloud in real time, with synchronized display of the manipulation video and communication audio of the grassroots doctors (Figure 1).

**Figure 2:**
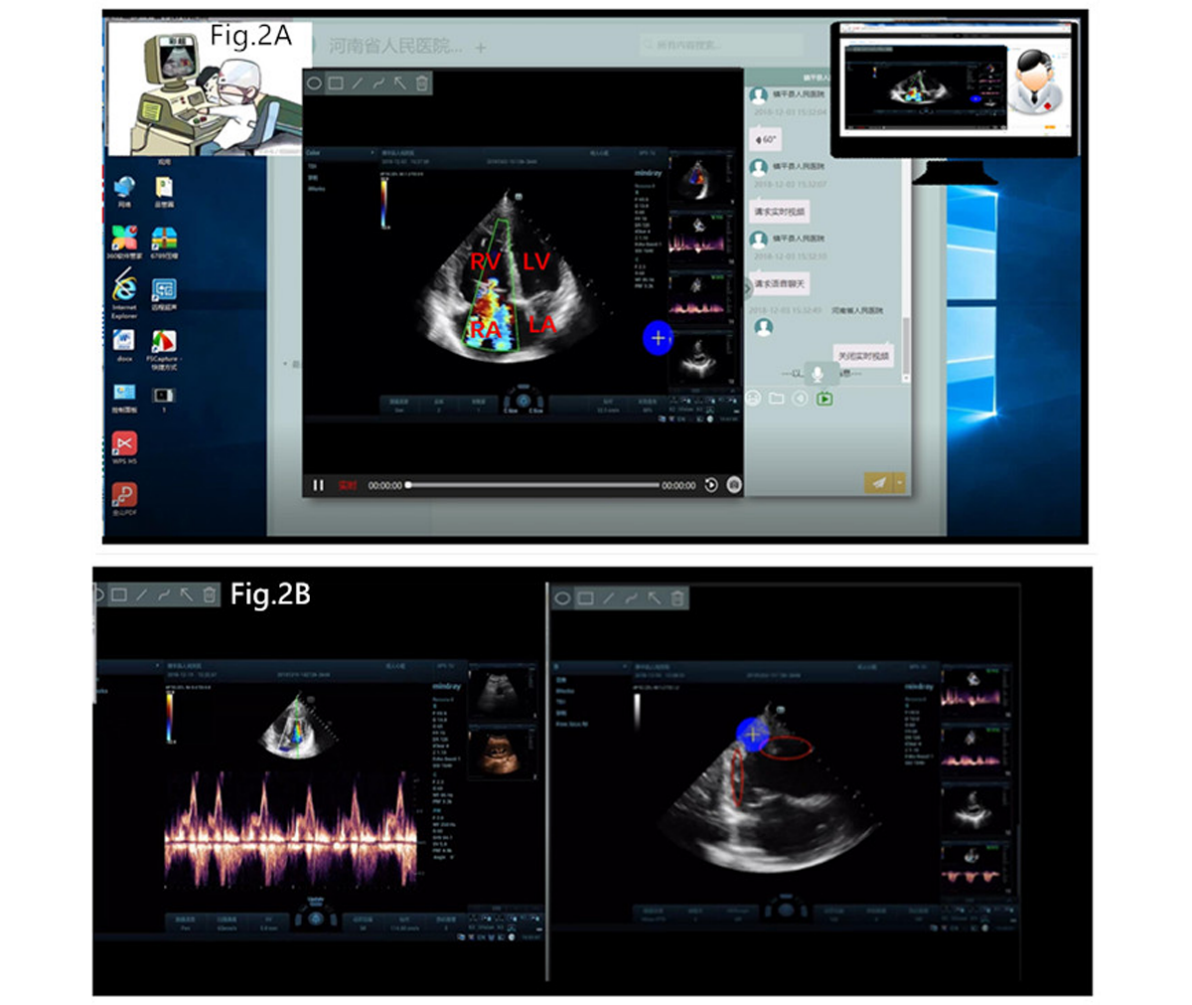
Pictures of remote transthoracic echocardiography consultation. Panel 2A shows the computer interface for remote consultation in one patient. The left upper corner shows an ultrasound physician at the grassroots hospital examining the patient; the right upper corner shows the scene of expert consultation at the superior hospital; in the middle is the image on the 4-chamber view in real time. Panel 2B shows the images of two patients in real time. The left upper corner of the interface is where the electronic marking tool can be found; both parties can add or delete electronic marks at any time, thus achieving three synchronization and double-real-time interaction. The red ellipse in the right insert is the electronic mark. LA: left atrium; LV: left ventricle; RA: right atrium; RV: right ventricle.

Consultation experts could watch and guide the manipulations of the grassroots doctors. Electronic marks could be added to or deleted from the TTE images by using the electronic marking tool, which made the system “interactive” and “real time.” These marks were useful for analyzing abnormal images and lesions, diagnosis, guidance and teaching, and quality control. In addition, the two parties could review the contents of consultation, and there was free switch between real-time broadcasting and off-line browsing. This offered a good choice for clinical teaching and technical training (Figure 2).

### Consultation Workflow

This system was supported by the internet smart hierarchical health care coordination platform of Henan Provincial People’s Hospital. Both the applying party and consulting party could set up log-in accounts and passwords. The applying party applied for remote TTE consultation on the platform after registration and log-in. The basic information of the patients was filled in, and after confirmation, the information was sent to the consultation experts in the form of text messages. The consulting party would log in, check the list of applications, be informed of the patients’ information, and consent to the consultation. After the consultation was over, the electronic consultation report sheet was filled and feedback was sent to the secondary care hospitals.

### Data Storage and Statistics

Remote TTE images were stored at the Henan Provincial People’s Hospital server with back-up, and the safety of the data was ensured. All statistical analyses were conducted using SPSS 18.0 software (SPSS Inc, Chicago, IL). Measurements were expressed as mean (SD).

## Results

A total of 45 patients, including 23 men and 22 women, were included in the study. The patients were aged 4 days to 90 years, with a mean age of 57.24 (SD 21.63) years.

All patients received synchronized real-time interactive remote TTE consultation. Through real-time audio interaction, the experts were fully informed of the patients’ medical history and examination results and provided standard guidance for the scan in each cardiac view. The purpose of this study was to improve the efficiency of consultation and increase the accuracy of consultation. Therefore, all patients had passed the preliminary examination, and all echocardiographic indicators were measured before consultation at the secondary care hospitals. The consultation experts also knew details of each patient in advance, including gender, age, history of present illness, previous history, electrocardiogram, and other imaging data. In the consultation process, experts focused on scanning the key sections and diagnosis of the disease, and each indicator was no longer detected unless necessary or there was doubt about the results. The total consultation time was approximately 341.31 min for 45 patients, and the average time for each patient was 7.58 (SD 6.17) min. A total of 75.6%, 22.2%, and 2.2% of patients had consultation times ≤10 min, 10-30 min, and >30 min, respectively. There was no significant correlation between the age of patients and consultation time (*P*>.05).

Of 45 patients receiving remote consultation, 3 were diagnosed with congenital heart diseases at the secondary care hospitals (7%) and 42 were diagnosed with acquired congenital heart diseases (93%) at the secondary care hospitals, which were caused after birth. After expert consultation, the final diagnosis was congenital heart disease in 5 patients (11%), acquired congenital diseases in 34 patients (76%), and absence of cardiac abnormalities in 6 patients (13%).

Compared with the initial diagnosis at the secondary care hospitals, seven patients (16%) were newly diagnosed with abnormalities of the heart after remote TTE consultation (Table 1). After expert consultation, a definite diagnosis was made in six patients (13%; Table 2). The initial diagnosis made by the secondary care hospitals was rejected by experts in six patients (13%). Among them, four patients were suspected of segmental ventricular wall abnormal motion upon initial diagnosis, one patient was suspected of left ventricular hypertrophy, and one patient was suspected of Kawasaki disease. For the remaining 26 patients (58%), the expert opinions agreed with the initial diagnosis at the secondary care hospitals. During consultation, the experts provided detailed answers to questions about some common and difficult clinical problems posed by the rural doctors. These questions were concerned with classification of atrial septal defect, influence of radiotherapy and chemotherapy on the heart, differentiation between degenerative changes of mitral annulus, differentiation between dilated cardiomyopathy and ischemic cardiomyopathy, assessment of pulmonary hypertension, and the relationship between cardiac blood supply regions and segmental ventricular wall abnormal motion.

Among 45 patients receiving remote TTE consultation, 3 patients were advised to receive surgery, 5 patients were advised to receive further examinations at tertiary care centers, and 5 patients were recommended regular re-examinations.

In addition to the abovementioned health care benefits, the patients saved the expenses of a round trip of travel. The average trip expense per time for each patient from Fan County People’s Hospital of Puyang City or Zhenping County People’s Hospital of Nanyang City to Henan Provincial People’s Hospital by common transportation vehicles was 100 RMB. For the 45 patients receiving remote consultation, at least 9000 RMB was saved for a round trip, and fess of accommodation, meals, and registration were not covered. Moreover, the cardiac abnormalities were excluded for six patients, which avoided unnecessary transportation and further examinations, further reducing health care expenses and costs for the patients.

**Table 1:** Comparison of diagnoses before and after expert consultation.

Initial diagnosis at the secondary care hospitals	New diagnosis by expert consultation
Dilated cardiomyopathy	Ischemic cardiomyopathy and segmental ventricular wall abnormal motion
Occlusion of ventricular septal defect	Partial noncompaction of the left ventricular myocardium
Dilated cardiomyopathy	Dilated cardiomyopathy and partial noncompaction of the left ventricular myocardium
Routine ultrasonography (left ventricular wall thickening; tachycardia; thickening of the basal interventricular septum)	Ventral septal defect (left ventricular wall thickening and ventricular septal defect; tachycardia and forward movement of the apex of the mitral valve at systole; thickening of the basal interventricular septum and mild pressure gradient in the left ventricular outflow tract)

**Table 2:** Information of patients confirmed after consultation.

Initial diagnosis at the secondary care hospitals	Confirmation by expert consultation
Complex congenital heart diseases	Double outlet of right ventricle, ventricular septal defect, and transposition of great arteries
Complex congenital heart diseases	Complete transposition of great arteries and ventricular septal defect
Rheumatic heart disease	Degenerative changes of mitral annulus
Strong echoes in the papillary muscles (pulmonary hypertension [severe]; dilated cardiomyopathy)	Papillary muscle calcification (pulmonary hypertension [moderate]; ischemic cardiomyopathy)

## Discussion

### Principal Results

Telemedicine has become a new hotspot for the development of network communication technology [8]. The United States is the first country that initiated telemedicine, followed by the United Kingdom, Japan, Mexico, Korea, and Europe. For example, the European Union launched a large-scale experiment of the telemedicine system that covered 3 biomedical engineering laboratories, 10 major companies, 20 pathology laboratories, and 120 terminal users to promote popularization of telemedicine [9].

China’s telemedicine service dated back to the 1980s. It is generally believed that China’s earliest telemedicine activity was the telegraph consultation of the acute disease of the oceangoing freighter conducted by Guangzhou Ocean Shipping Corporation in 1986 [10]. China’s first telemedicine activity in modern times was the remote discussion of neurosurgery in one case between the PLA General Hospital and a hospital in Germany in 1988 [11]. Along with the rapid progress in internet technology, many large hospitals in different parts of China have established remote consultation centers in the 21st century [4,12]. Thus far, the development is more successful in terms of remote consultation of specific cases, remote imaging, remote echocardiogram, and remote pathological diagnosis. Remote ultrasonography, especially remote TTE consultation and diagnosis, is less developed, which is mainly because it is an incomplete technology in dynamic image coding and decoding with multisource information synchronization and low network transmission speed.

By using the cloud computing platform coupled to unique dynamic image coding and decoding technology, multidimensional communication information in the form of voice, texts, and pictures is integrated. A remote TTE consultation system connecting Henan Provincial People’s Hospital and two secondary care hospitals 300 km away was built in this study. This system was then applied in consultation for the 45 patients at the secondary care hospitals and achieved satisfactory effects. Under the remote TTE consultation, new lesions were found in seven cases (16%) by the consultation experts, the initial diagnosis was confirmed in six cases (13%), and the initial diagnosis was denied in six cases (13%). In addition, some questions about difficult and rare illnesses were answered by the physicians at the tertiary care center. The satisfaction rate was 100% for both patients and doctors. Among them, three patients were advised to undergo surgery, five patients were advised to undergo further examinations at tertiary care centers, and five patients were recommended regular re-examinations. The total consultation time was approximately 341.31 min for the 45 patients, and the mean time for each patient was 7.58 (SD 6.17) min.

### Comparison With Prior Work

In recent years, internet-based remote TTE consultation has been reported. A primary health care center in Sweden has achieved long-distance image transmission via a robot arm and an electronic health program. Cardiologists at tertiary care centers are invited for consultation, and some positive preliminary results are available. However, the sample size is small, and the feasibility of such consultation remains to be further verified [13]. Korean physician Changsun Kim has attempted to use a network video telephone technology based on a smartphone, through which the technicians at secondary care hospitals are given guidance on TTE and observe left ventricular ejection fraction. The usefulness of such technology is limited for secondary care hospitals, mainly due to image transmission quality, illumination intensity, and mobile phone performance [14]. American scholars Rouse et al [6] reported an asynchronous trans-Pacific remote TTE diagnosis; this method effectively reduced the risk of long-distance trans-Pacific transfer of patients and the costs. However, there were also problems with this approach, as it was not in real time, lacked quality control, and was time consuming [6]. In recent years, some scholars have made some remarkable innovations in the methodology and efficiency of cloud-based remote TTE consultation and diagnosis [15], although such technology is still restricted by the bandwidth. In 2013, a multicenter study led by physicians Webb et al in Washington DC indicated that the median time for diagnosis was 100 (SD 67) min (range: 10-311 min) for telemedicine [7]. Compared with the previous reports, the remote TTE consultation platform in our study greatly reduced the consultation time and improved the diagnostic efficiency. With the synchronization of dynamic ultrasound images, audio, and video, the consultation experts can guide echocardiographers at the secondary care hospitals. Thus, the effect of real-time transmission and interaction is achieved, which is conducive to improving the skills of echocardiographers at the secondary care hospitals.

The remote TTE consultation system has the following benefits: First, the precision of multichannel signal synchronization is below 3 ms. In other words, the delay time of information transmission between the two hospitals does not exceed 3 ms, which can truly achieve real-time transmission of ultrasonic images, video, and audio. Second, only simple hardware and equipment are required for this system. Through this system, the consultation physicians at tertiary care centers can not only guide manipulation, but also direct diagnosis in real time. In addition, using the real-time interactive system, experts can be engaged in direct communication with the patients.

### Limitations

Despite the abovementioned advantages, this platform needs improvement. First, the stored images cannot be remeasured or processed by consultation physicians at the tertiary care centers. Second, the structured electronic report remains to be further developed so that is can be legally used. Third, the introduction of a voice-recognition system led to smarter and more convenient generation of an electronic report, reduced errors caused by manual input, and further improved the quality of remote consultation.

### Conclusions

In summary, the synchronized real-time interactive remote TTE consultation system based on a cloud service is featured by simple equipment, convenient connections, easy implementation at a small bandwidth, clear images, and easy operation.

## Abbreviations

HDMI: high-definition multimedia interface
TTE: transthoracic echocardiography
